# Glass-Forming Ability and Soft Magnetic Properties of (Co_75_Ti_25_)_100−x_Fe_x_ (x; 0–20 at.%) Systems Fabricated by SPS of Mechanically Alloyed Nanopowders

**DOI:** 10.3390/nano10050849

**Published:** 2020-04-28

**Authors:** Mohamed Sherif El-Eskandarany, Naser Ali, Maryam Saeed

**Affiliations:** Nanotechnology and Applications Program, Energy and Building Research Center, Kuwait Institute for Scientific Research, Safat 13109, Kuwait

**Keywords:** mechanical alloying, high-energy ball-milling, solid-state reaction, solid-solution, layered structure morphology, phase transformation, glass-transition, crystallizations, supercooled liquid region, thermodynamics

## Abstract

Due to their outstanding mechanical properties and soft magnetic characteristics, cobalt-based metallic glassy alloys have stimulated much interesting research. These metastable ferromagnetic materials possess very small magnetocrystalline anisotropy, and almost zero magnetostriction. They reveal low coercivity, extremely low core loss, moderate saturation polarization, and very high magnetism. Despite these attractive physical behaviors, Co-based metallic glasses are difficult to obtain by the melting/casting and conventional rapid solidification techniques due to their poor glass-forming ability. In the present study, we succeed in preparing (Co_75_Ti_25_)_100−x_Fe_x_ (x; 0–20 at.%) metallic glassy powders, using a mechanical alloying approach. The end product of the as-prepared powders was consolidated into full dense cylinders with large-diameter and thickness (2 × 2 cm), using spark plasma sintering technique. The results have shown that the consolidation step did not lead to any undesired crystallizations or phase transformations, and the as-consolidated buttons maintained their unique short-range order structure. These bulk metallic glassy systems possessed high glass-transition and crystallization temperatures, suggesting their high thermal stability. However, they showed low values of the reduced glass-transition temperatures, indicating that this system is difficult to prepare by the conventional way of preparations.

## 1. Introduction

### 1.1. Background

With reference to the atomic arrangement of solid materials, atoms are arranged either in long-range order (crystalline materials) or in short-range order (amorphous/glassy materials) fashions [1,2,3]. Metallic glassy alloys date to 1960, when Duwez and his group discovered the possibility of preparing Au_80_Si_20_ metallic glass, using a rapid quenching technique [4]. Due to their exciting unique properties, bulk metallic glasses (BMG) have attracted many metallurgists and materials scientists to investigate an uncounted number of metallic glassy systems over the last 6 decades. Pd_77.5_Cu_6_Si_16.5_ [5], Pd–T–P alloys (T = Ni, Co, Fe) [6], Pd–Ni–P [7], La–Al–Ni and La–Al–Cu alloys [8], Al-Ta alloys [9], Zr–Ti–Cu–Ni–Al alloys [10], Cu_33_Zr_67_ [11], and Ti_40.6_Cu_15.4_Ni_8.5_Al_5.5_W_30_ alloy [12] are a few typical examples of metallic glassy systems that have been prepared since 1960. All work related to the preparations, characterizations, and implementations of metallic glassy alloys has discovered advanced properties of this category of materials that are not found when the same alloys are in their stable crystalline states [13]. The recent studies of a wide range of new metallic glassy systems have discovered beneficial properties of these materials, such as unusual corrosion resistance, high hardness, unique mechanical ductility, and yield strength, and high magnetic permeability with low coercive force [14,15,16,17,18,19,20]. Therefore, metallic glasses are now considered to be promising candidates for several technical applications in engineering, electronics, medical, defense and aerospace industries, and sports sectors [15,21,22,23,24,25,26,27,28].

### 1.2. Metallic Glassy Soft Magnetic Materials

In recent years, metallic glassy soft magnetic materials, in particular Co-based BMG have stimulated much interesting research. This is attributed to their ultrahigh strength and excellent soft magnetic behaviors [17,20,29,30,31,32]. In fact, ferromagnetic Co-metallic glassy alloys possess very small magnetocrystalline anisotropy, as well as (near) zero magnetostriction [20], which is responsible for their exceptional soft magnetic behaviors, indexed by very low coercivity, extremely low core loss, moderate saturation polarization, and very high magnetism [33]. Based on these excellent characteristics, Co-based metallic glasses have become the best choices for several electromagnetic industrial applications, such as pulse compression applications, flexible antenna, keyless entry systems, power and current transformers, spike blockers, magnetic shields, and sensors [20]. In addition, Co-based metallic glassy alloys offer the opportunity to decrease transformer core losses [31]. In particular, a large elastic flexibility guarantees excellent insensitivity with respect to plastic deformations and a small electrical conductivity reduces the eddy–current losses [34].

### 1.3. Aim of the Present Work

The present study has been undertaken in part to fabricate metallic glassy-(Co_75_Ti_25_)_100−x_Fe_x_ (x; 0–20 at.%) nanopowders via mechanical alloying (MA) technique, using high-energy ball-milling (BM). This study aims to prepare BMG-(Co_75_Ti_25_)_100−x_Fe_x_ ternary systems upon consolidation of the BM powders into full dense bulk material, using spark plasma sintering (SPS) technique.

Because of their poor glass-forming ability and the absence of any deep eutectic compositions in the equilibrium phase diagram, it is very difficult to prepare BMG-Co-based alloys with large dimensions. Most of these alloys can be only produced in the form of ribbons and/or as very small rods with diameter of 3 mm [20]. Furthermore, the glass-forming ability of this new ternary system has been investigated as a function of Fe-metal additives in the range between 0 at.% to 20 at.%. In addition, the effect of Fe concentration on the thermal stability and magnetic properties of Co-Ti base is reported.

## 2. Materials and Methods

### 2.1. Starting Feedstock Materials

Pure elemental powders of Co (150 μm, >99.9 wt.%, #266647-Sigma–Aldrich, St. Louis, MO, USA), Ti (45 μm, >99.99 wt.%, #366994 Sigma–Aldrich), and Fe (45 μm, >99 wt.%, #12310 Sigma–Aldrich) were used as the starting feedstock materials. The powders were handled, balanced, and then mixed inside He-atmosphere (99.99%) glove box (UNILAB Pro Glove Box Workstation, mBRAUN, München, Germany) to get an amount of 25 g with the desired nominal composition of −(Co_75_Ti_25_)_100−x_Fe_x_ (x; 0–20 at.%). Detailed ICP analysis of the starting composition, given in at.% and wt.% is presented in Table 1.

### 2.2. Preparations of Metallic Glassy Alloy Powders

The mixed powders of each composition were individually charged into tool steel vials (500 mL in volume) and sealed together with 75 tool steel balls (11 mm in diameter) in the glove box, using a ball-to-powder weight ratio of 20:1. The vials were then maintained on a high-energy ball mill (Planetary Mill PULVERISETTE 5, Fritsch, Germany), where the BM process was carried out for 15, 30, 45, and 60 h at an ambient temperature. After each milling run, a small amount (<500 mg) was discharged from the vial for different analysis. Then, the BM process was resumed under the same operating conditions.

### 2.3. Powder Consolidation by Spark Plasma Sintering (SPS)

The powders obtained after 60 h of BM were individually consolidated into dense buttons, using the SPS (Dr Sinter Lab. Instrument, Fuji, Japan) technique. The SPS system consisted of a sintering press unit with a vertical single-axis pressurization, special designed punch electrodes incorporating a water cooler, a water-cooled vacuum chamber, a vacuum/air/argon-gas atmosphere control mechanism, a special (direct current) DC pulse-sintering power generator, a cooling-water control unit, *Z*-axis position-measuring and control unit, temperature-measuring and control units, an applied pressure display unit, and various safety interlock devices.

In this study, the as-BM powders were charged into a graphite die and stacked between upper and lower punches. To ensure an easy ejection of the sample after sintering and in order to avoid any reactions between the internal surfaces of the die tools (die and punches) with the powders (sample), graphite sheets are used as spacers. For reducing the amount of radiant heat transfer to the machine, the entire die and punch assembly were wrapped with carbon felt, which is held closed using carbon yarn. The die was then mounted on the sintering stage inside the SPS chamber and held between upper punch and lower punch electrodes. During the SPS process, temperature was set far below the crystallization temperature of the metallic glassy powders of each composition. A pyrometer was used to measure the temperature of the die surface during sintering process.

In the present work, the powder sintering with SPS process was conducted by the presence of an electric field, known as field assisted sintering technique (FAST). In contrast with the conventional hot-pressing technique, in which the sample is heated from the outside, the sintering procedure in SPS took place upon heating the sample internally by the passage of an electric current with extremely high heating and cooling rates of 580 and 280 K per min, respectively. The external pressures applied during the sintering process was in the range of 10–15 MPa. The whole process, including temperature ramp and holding times, was about 6 min.

### 2.4. Sample Characterizations

#### 2.4.1. Crystal Structure

The crystal structures of all samples were investigated by X-ray diffraction (XRD) with CuKα radiation, using 9 kW Intelligent X-ray diffraction system, provided by SmartLab–Rigaku, Tokyo, Japan. The local structure of the synthesized materials was studied by 200 kV-field-emission high-resolution transmission electron microscopy/scanning transmission electron microscopy (HRTEM/STEM) supplied by JEOL-2100F, Tokyo, Japan, and equipped with Energy-dispersive X-ray spectroscopy (EDS) supplied by Oxford Instruments, UK. Cryo Ion Slicer Machine (IB-09060CIS) supplied by JEOL-2100F, Japan was used to prepared bulk TEM samples of as-SPS buttons.

#### 2.4.2. Morphology and Elemental Analysis

The morphological characteristics of the milled and consolidated samples were investigated by means of field-emission scanning electron microscope (FE-SEM), using 15 kV JSM-7800F, JEOL-Japan. The local elemental analysis was investigated by EDS, Oxford Instruments-UK) system interfaced with the FE-SEM.

#### 2.4.3. Thermal Stability

Differential scanning calorimeter (DSC), provided by Setaram, Caluire-et-Cuire, France, using a heating rate of 40 °C/min, was employed to investigate the glass-transition temperature, glass-forming ability, and thermal stability indexed by the supercooled liquid region and crystallization temperature of the metallic glassy samples.

#### 2.4.4. Density Measurements

The density of the consolidated samples was measured by the Archimedean approach, using toluene.

#### 2.4.5. Magnetization Measurements

The polarization magnetization (Bs) of the as-consolidated samples was measured at room temperature, using a vibrating sample magnetometer (VSM) with a maximum applied magnetic field of 670 kA/m. The coercive force was measured with a B-H loop tracer.

## 3. Results and Discussion

### 3.1. Ball-Milling Effect on Structure and Morphology of the Mechanically Alloyed Powders

**Figure 1:**
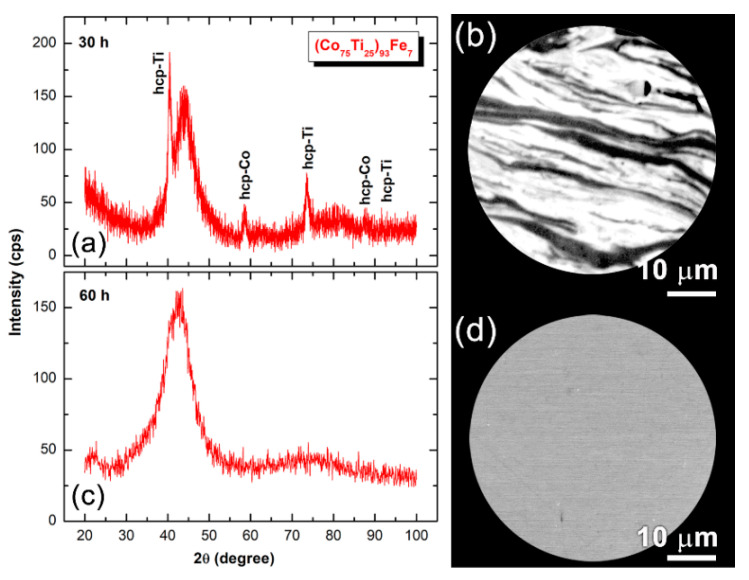
XRD patterns of MA-(Co_75_Ti_25_)_93_Fe_7_ powders obtained after 30 h of BM and FE-SEM micrographs of cross-section view are presented in (**a**) and (**b**), respectively, where (**c**) and (**d**) display the XRD and FE-SEM of cross-section view for the powders milled for 60, respectively.

Figure 1a displays the XRD pattern of MA-(Co_75_Ti_25_)_93_Fe_7_ powders obtained after BM for 30 h. After this stage of MA, the XRD revealed a broad diffuse halo of an amorphous phase coexisted with unprocessed hcp-Ti, and –Co crystals, as shown in Figure 1a. The FE-SEM of cross-section view of the powders milled for 30 h exhibited intimated lamella corresponding to the diffusion couples of metallic elements, as presented in Figure 1b. After 60 h of MA time, all the Bragg peaks corresponding to pure Co and Ti elements disappeared, where the halo-peak had become pronounced, indicating the compilation of MA process and formation of an amorphous structure phase (Figure 1c). The formed amorphous phase coexisted with ultrafine nanocrystalline particles of unprocessed Fe-metal, as demonstrated by the sharpness seen in the first primary peak (Figure 1c). In contrast to the layered-like morphology shown in Figure 1b representing the 30 h MA sample, contrast-free mirror-like morphology was obtained for the powders milled for 60 h, indicating the absence of crystalline phase (Figure 1d).

**Figure 2:**
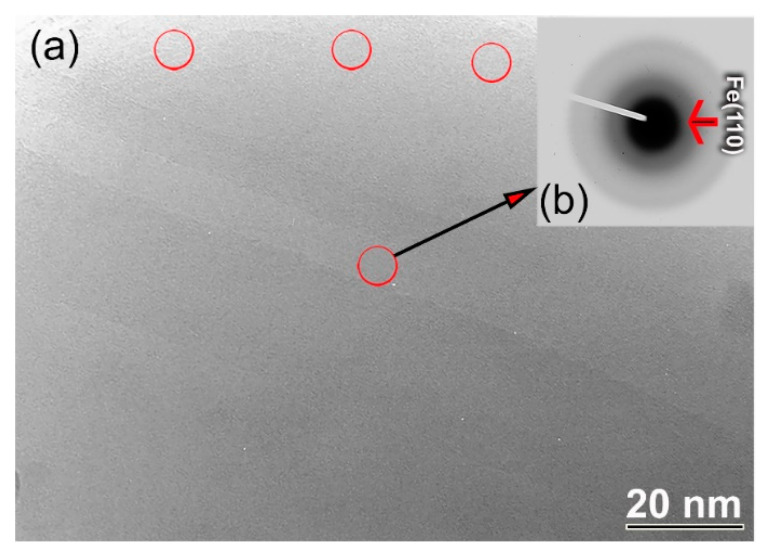
(**a**) BFI and (**b**) SADP of MA-(Co_75_Ti_25_)_93_Fe_7_ powders obtained after 60 of BM.

This sample exhibited a featureless fine structure beyond the sub-nano level, as implied by the bright field image (BFI) shown in Figure 2a. However, nano-dimensional lenses of a crystalline phase existed in some areas, as indexed by the circular symbols shown in Figure 2a. The selected area diffraction pattern (SADP) that was taken from the center of Figure 2a indicates the formation of an amorphous structure, implied by the spot-free halo-diffuse pattern. However, these halo-diffuse rings overlapped with a rather sharp ring related to nanocrystalline Fe (110), as displayed in Figure 2b.

**Figure 3:**
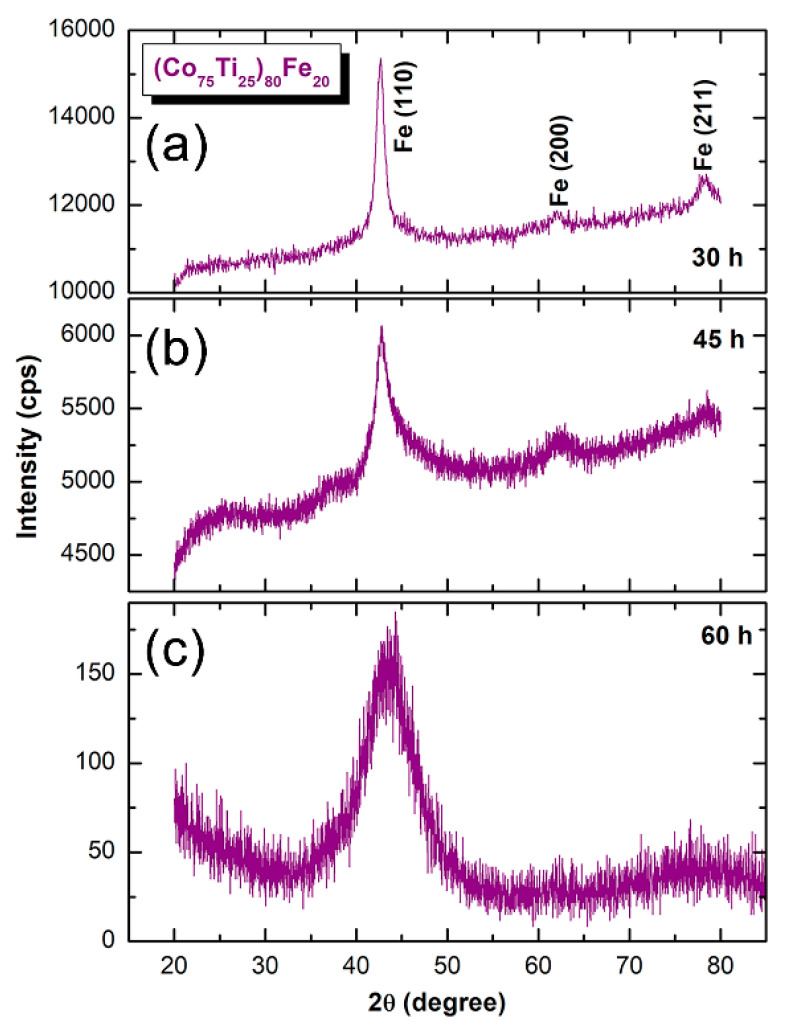
XRD patterns of MA-(Co_75_Ti_25_)_80_Fe_20_ powders obtained after (**a**) 30, (**b**) 45, and (**c**) 60 h.

Surprisingly, the XRD pattern of Fe-rich side for MA-(Co_75_Ti_25_)_80_Fe_20_ powders obtained after BM for 30 h displayed only Bragg peaks corresponding to bcc-Fe, where the Bragg lines related to hcp-Ti and –Co are hardly detected, as presented in Figure 3a. This may suggest the formation of bcc-FeCoTi solid-solution phase. As the MA time increased (45 h), the Bragg peaks related to the formed bcc-metastable phase had become wider, suggesting the progress of grain refining, as presented in Figure 3b.

**Figure 4:**
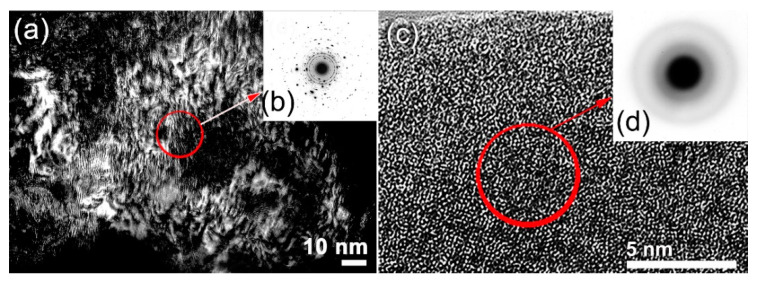
(**a**) DFI and (**b**) corresponding SADP of MA-(Co_75_Ti_25_)_80_Fe_20_ powders milled for 45 h. Elsewhere, the atomic-resolution FE-HRTEM image and the its related NBDP of MA-(Co_75_Ti_25_)_80_Fe_20_ powders obtained after 60 of BM are presented in (**c**) and (**d**), respectively.

The dark field image (DFI) and SADP of the powders milled for 45 h are shown in Figure 4a,b, respectively. The powders of this obtained bcc-solid-solution were severely plastically deformed due to the milling process (Figure 4a). This is demonstrated by the formation of an intensive “network” of lattice imperfections composited of dislocations, point and lattice defects. The local structure of the powders after this milling stage did not indicate the formation of an amorphous phase, as implied by the spot-like SADP (Figure 4b). Increasing the BM time enhanced the mechanical imperfections, leading to the conduction of a solid-solution-to-amorphous phase transformation upon BM for 60 h, as suggested by manifesting of a halo-diffuse diffraction pattern without evidence of existing of Bragg lines related to the solid-solution phase (Figure 3c). A complete bcc-solid-solution-to-amorphous phase transformation was confirmed by FE-HRTEM, which shows a dense random close-packed structure with maze-like morphology (Figure 4c). The related nano-beam diffraction pattern (NBDP) exhibited amorphous-like halo-diffuse structure, as shown in Figure 4d.

**Figure 5:**
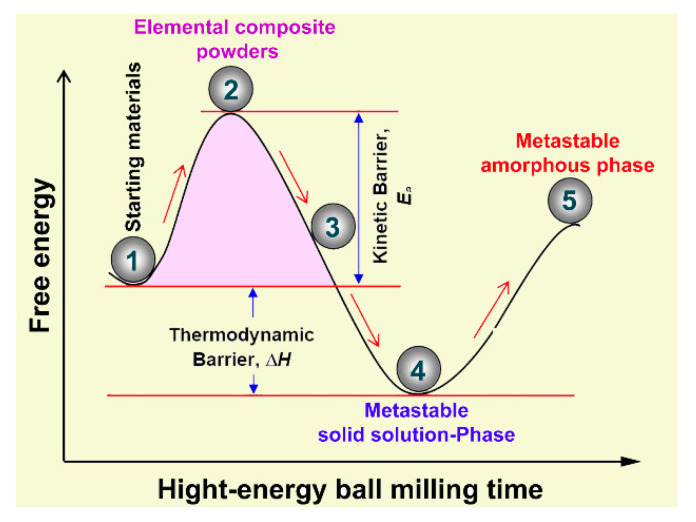
Schematic of potential mechanism proposed for describing the solid-solution-to-amorphous phase transformation taken place ternary (Co_75_Ti_25_)_93_Fe_7_ system upon increasing the BM time.

Figure 5 summarizes the mechanism taken place upon high-energy BM of ternary (Co_75_Ti_25_)_93_Fe_7_ powders. At the early stage of milling, the feedstock elemental powders (point 1) tended to form agglomerated powders (point 2). These aggregated powder particles were heavily subjected to mechanical deformation upon increasing the BM time. Introducing such lattice defects enhanced the solid-state reaction between the alloying elements of Co, Ti, and Fe metals to form a reacted bcc-metastable phase coexisted with unprocessed elemental powders, as referred by point 3 in Figure 5. Furthermore, longer BM time led to the stabilization of the solid-solution phase, reaching to larger negative value of free energy (ΔG), as denoted by point 4. Since this formed solid-solution product is in metastable phase, it failed to withstand against the plastic deformation, shear stresses, and lattice defects introduced to the powders by the BM media with longer milling times. As a result, it was gradually losing free energy and eventually transformed to a less stable amorphous phase (point 5) with the same composition.

**Figure 6:**
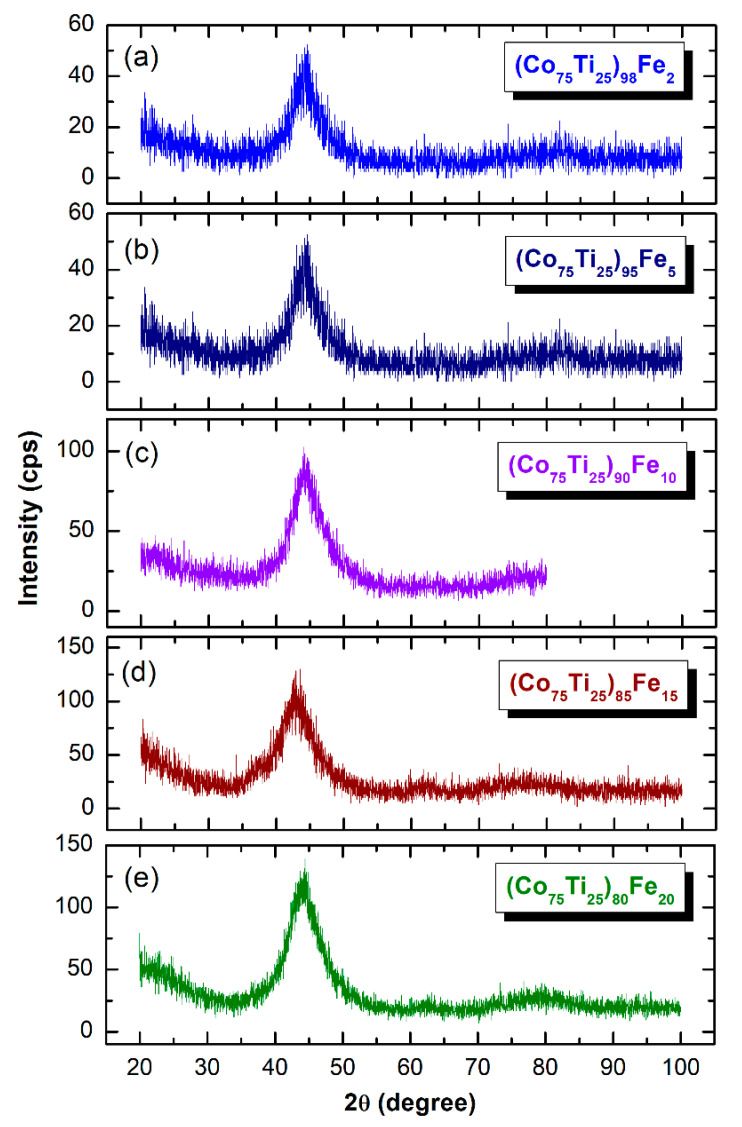
XRD patterns of MA (**a**) (Co_75_Ti_25_)_98_Fe_2_, (**b**) (Co_75_Ti_25_)_95_Fe_5_, (**c**) (Co_75_Ti_25_)_90_Fe_10_, (**d**) (Co_75_Ti_25_)_85_Fe_15_, and (**e**) (Co_75_Ti_25_)_80_Fe_20_ powders, obtained after 60 h of BM time.

The XRD patterns of MA-(Co_75_Ti_25_)_100−x_Fe_x_ (x; 2, 5, 10, 15, and 20 at.%) obtained after 60 h of BM time are displayed together in Figure 6. All the XRD patterns exhibit only broad diffuse haloes, in scattering range of 2θ between 40–50°, with existence of minor volume fractions of unprocessed Fe-nanoparticles, as demonstrated by the sharpness shown in the first primary halo patterns. The irregularity shown in the base line may be attributed to the surface oxidation of the powders during the XRD analysis. This implies the capability of an MA approach to fabricate the desired amorphous alloy powders in wide amorphous range.

**Figure 7:**
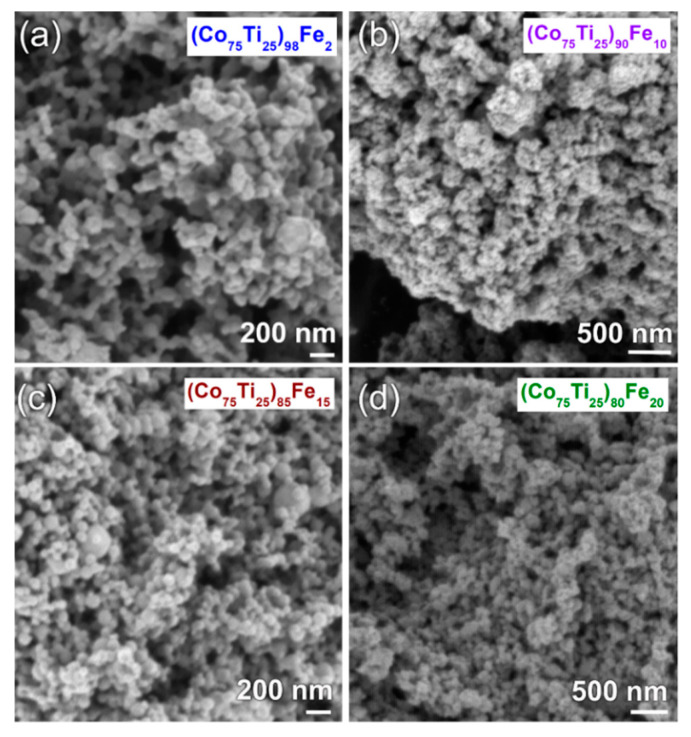
High-magnification FE-SEM micrographs of as-MA (**a**) (Co_75_Ti_25_)_98_Fe_2_, (**b**) (Co_75_Ti_25_)_90_Fe_10_, (**c**) (Co_75_Ti_25_)_85_Fe_15_, and (**d**) (Co_75_Ti_25_)_80_Fe_20_ powders, obtained after 60 h of BM time.

The high-magnification FE-SEM micrographs of selected MA systems obtained after 60 h of BM time are shown in Figure 7. In general, the as-MA powders consisted of ultrafine nanoparticles with particle size ranging between 80 nm to 120 nm, as displayed in Figure 7. These spherical powder nanoparticles tended to form aggregates due to the Van der Waals and electrostatic effects (Figure 7). In all systems, the as-fabricated amorphous powders had a narrow size particle distribution range, as demonstrated in Figure 7. It has been pointed out by Diouf et al. [35] that powder consolidation by SPS is affected with their sizes. Finer spherical powders that possess great contact points and neck formation lead to a successful consolidation process. Liang and Jin [36] have also studied the effect of powder particles sizes on SPS process. They concluded that the fine spherical powders were beneficial for fabricating dense bulk materials via SPS approach.

### 3.2. Consolidation of as-MA Powders into BMG by SPS Technique

**Figure 8:**
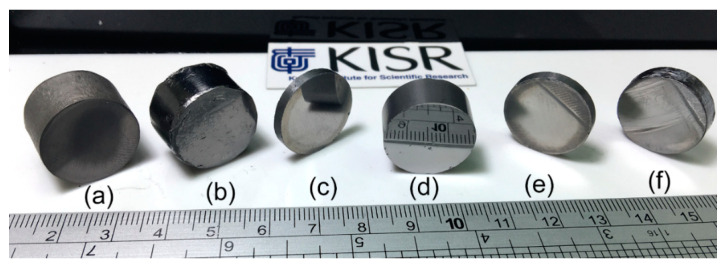
A general photo presenting a macrograph of selected BMG systems of (**a**) (Co_75_Ti_25_)_98_Fe_2_, (**b**) (Co_75_Ti_25_)_95_Fe_5_, (**c**) (Co_75_Ti_25_)_90_Fe_10_, (**d**) (Co_75_Ti_25_)_85_Fe_15_, (**e**) (Co_75_Ti_25_)_80_Fe_20_, and (**f**) (Co_75_Ti_25_)_80_Fe_25_ consolidated buttons, obtained by SPS the as-MA powders for 60 h.

SPS technique was employed to consolidate the as-prepared amorphous powders obtained after 60 h of MA time into BMG. Neither cracks nor pores can be seen in the sintered samples (Figure 8), indicating a successful SPS procedure conducted to obtain nearly full dense BMG with metallic luster and smooth surface (Figure 8).

#### 3.2.1. Structural and Morphological Characteristics

**Figure 9:**
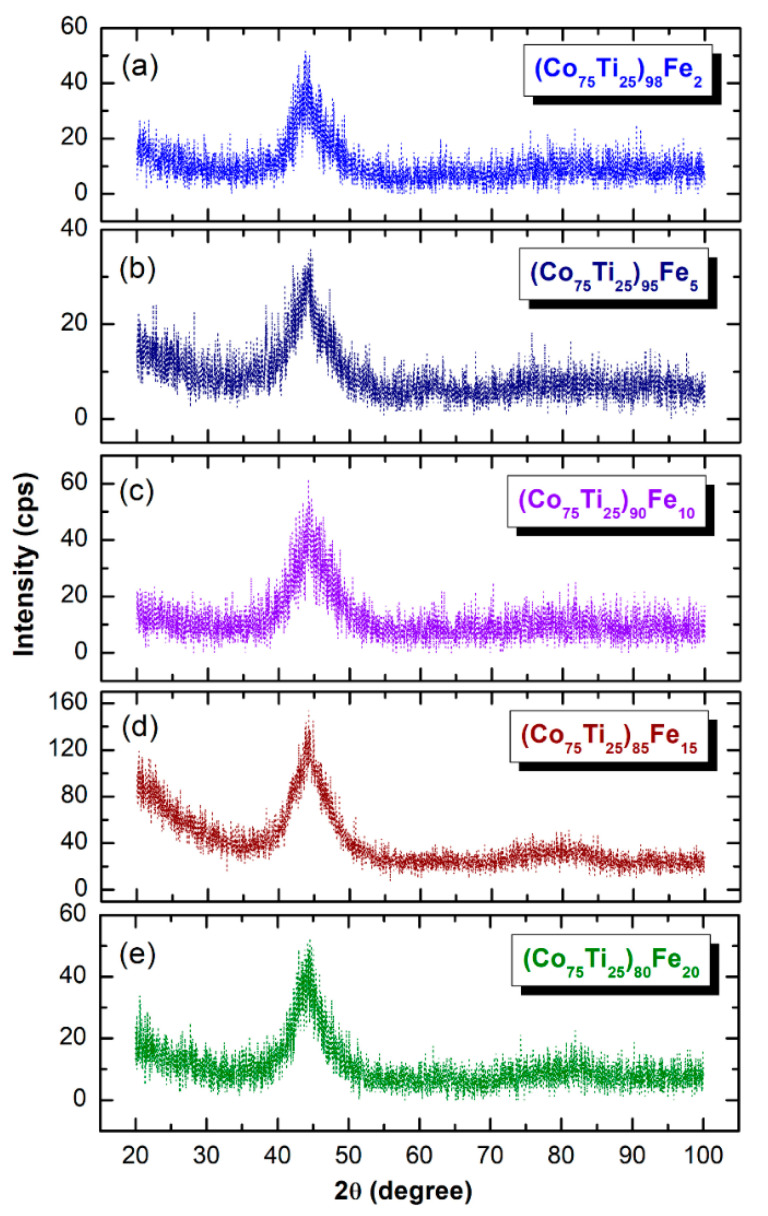
XRD patterns of as-MA for 60 h and then SPS buttons of (**a**) (Co_75_Ti_25_)_98_Fe_2_, (**b**) (Co_75_Ti_25_)_95_Fe_5_, (**c**) (Co_75_Ti_25_)_90_Fe_10_, (**d**) (Co_75_Ti_25_)_85_Fe_15_, and (**e**) (Co_75_Ti_25_)_80_Fe_20_ BMG system.

XRD, SEM, and TEM techniques were employed to ensure the capability of SPS for conducting successful consolidation process of amorphous powders without leading to undesired crystallization. Figure 9 displays the XRD patterns of as-consolidated amorphous powders, using SPS technique. Obviously displayed, all the XRD patterns without exceptions revealed broad diffuse halo-peaks existed with unprocessed Fe-nanoparticles (Figure 9). Comparing the XRD patterns presented in Figure 9 with the XRD patterns of as-prepared amorphous powders (Figure 1, Figure 3c and Figure 6), it can be concluded that the SPS step maintained the original short-range order structure of the amorphous powders without phase transformation.

**Figure 10:**
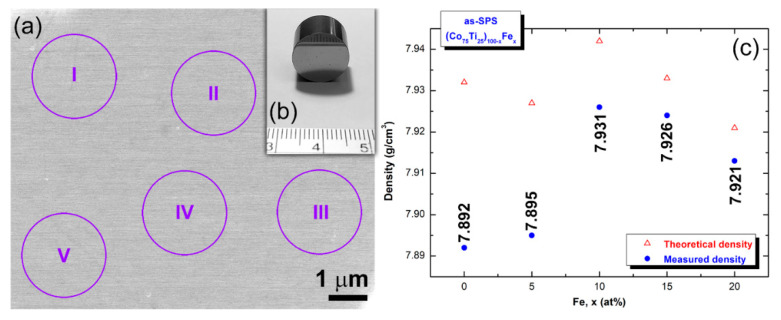
(**a**) FE-SEM micrograph of the cross-section view taken for a polished-mirror surface of consolidated (Co_75_Ti_25_)_80_Fe_20_ button (**b**). The EDS elemental analysis for the zones indexed in (**a**) are listed in Table 2. The theoretical and measured densities the consolidated buttons are shown in (**c**).

Figure 10a displays a typical FE-SEM micrograph of the cross-section view taken for a polished surface of consolidated (Co_75_Ti_25_)_80_Fe_20_ button (Figure 10b). No indication for existence of grains, or grain boundaries related to crystalline phase could be detected on the entire cross-section of this sample, implying that the consolidated button was amorphous phase. To understand the elemental distribution in this sample and to realize its homogeneity beyond the micro level, 5 individual zones (zones I to V) were selected when performing X-ray EDS elemental analysis (Table 2). The results indicated that the sample possesses excellent elemental distribution without compositional fluctuations or degradations, as shown in Table 2.

Figure 10c presents the theoretical and measured bulk densities of SPS-(Co_75_Ti_25_)_100−x_Fe_x_ buttons. SPS. All consolidate buttons had high values of relative densities, in the range between 99.5% to 99.8%, as shown in Figure 10c. The SPS sintering and sinter bonding of the nanopowders were achieved at low temperatures within a short period. This consolidation processes are conducted by charging the intervals between powder particles with electrical energy and effectively applying a high-temperature spark plasma generated at an initial stage of energizing momentarily, and an electromagnetic field and/or joule heating by continuous ON–OFF DC pulsed high electric current with a low voltage. One merit of the SPS consolidation step is that it maintains the original short-range order structure with no effect on any partial crystallizations.

**Figure 11:**
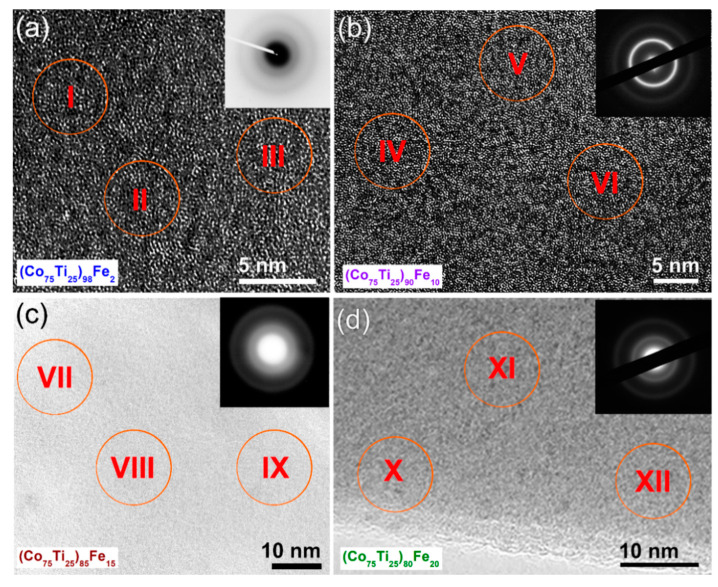
(**a**–**d**) FE-SEM micrograph of the cross-section view taken for a polished-mirror surface of consolidated (Co_75_Ti_25_)_80_Fe_20_ button (Figure 10b). The EDS elemental analysis for the zones indexed in (**a**) are listed in Table 2.

A cryo ion slicer process was used to prepare thin rings of 5 mm in diameter with thickness of less than 100 nm to perform HRTEM analysis for the as-consolidated buttons. Figure 11 shows the HRTEM micrographs and NBDPs of selected BMG consolidated systems, using SPS technique. All the samples revealed maze-like morphology of amorphous structure, characterized by the absence of lattice fringes beyond the nanolevel, as displayed in Figure 11. Moreover, the NBDPs displayed inset of the micrograph for each amorphous system indicate the formation of amorphous phase, as characterized by their sport-free haloes. Moreover, EDS results of the elemental analysis taken for those zones indexed in Figure 11 are presented in Table 3. The analysis implied that the SPS technique maintained the homogeneity of the chemical composition without fluctuation or degradation, as can be realized from Table 3. We can claim that SPS consolidation technique can be successfully used to consolidate amorphous materials without causing any undesired crystallization or formation of undesired phases.

#### 3.2.2. Magnetic Properties

**Figure 12:**
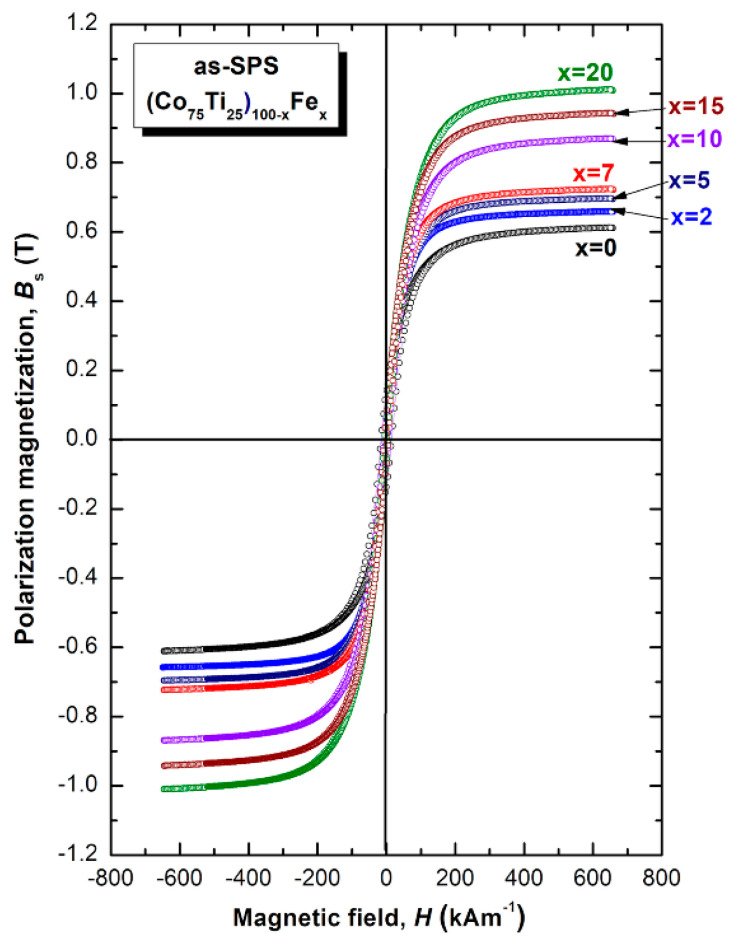
Hysteresis B-H loops of as-SPS (Co_75_Ti_25_)_100−x_Fe_x_ BMG materials.

The saturation magnetization (B_s_) and magnetic field (H) of SPS-(Co_75_Ti_25_)_100−x_Fe_x_ amorphous alloys were obtained according to the measured hysteresis loops, as depicted in Figure 12. The loops of all samples exhibited typical soft magnetic behaviors, as can be realized from Figure 12. The dependence of B_s_ on the Fe (x content) is clearly displayed. A pure binary Co_75_Ti_25_ amorphous system with (0 at.% Fe) revealed a rather modest value of B_s_ (0.61 T); however, when a small molar fraction of Fe (2 at.%) was alloyed with this binary system, the B_s_ increased to 0.70, as displayed in Figure 12. Further increase in the Fe content (2, 5, 7 at.%) led to improve the B_s_, depicted in Figure 12. This value reached to 0.72 T upon adding 7 at.% Fe. On the Fe-rich side (10 at.%) the B_s_ jumbled to 0.87 T (Figure 12), while it tended to increase upon adding 10 at.% Fe to reach to a higher value, as high as 0.94, as manifested by Figure 12. This value was continued to increase (1.01 T) upon increasing the Fe content (20 at.%), implying a rough linear correlation between Fe concentration and Bs.

#### 3.2.3. Thermal Analysis

DSC technique was used to characterize the crystallization behavior of as-SPS (Co_75_Ti_25_)_100−x_Fe_x_ amorphous alloys, indexed by their glass-transition temperature (T_g_), crystallization temperature (T_x_), supercooled liquid region (ΔT_x_ = T_x_ − T_g_), and enthalpy change of crystallization (ΔH_x_). In parallel, Differential thermal analysis (DTA) technique was employed to investigate their corresponding melting behaviors, characterized by melting temperature (T_m_), liquids temperature (T_l_), and reduced glass-transition temperature (T_rg_ = T_g_/T_l_).

**Figure 13:**
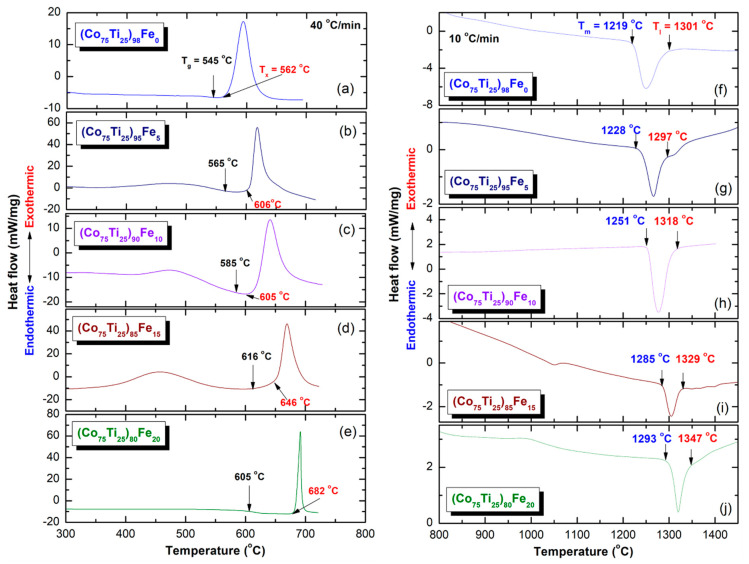
The DSC thermograms and corresponding DTA curves for SPS-(Co_75_Ti_25_)_100−x_Fe_x_ system are presented for each glassy alloy in (**a**–**e**) and (**f**–**j**), respectively. The onset temperatures related to T_g_, T_x_, T_m_, and T_l_ are indexed in each curve.

Figure 13a–e present the DSC curves of selected SPS-(Co_75_Ti_25_)_100−x_Fe_x_ samples, while their corresponding DTA traces are displayed in Figure 13f–j. The heating rates used for conducting DSC experiments was 40 °C/min, where it was 10 °C/min for DTA experiments. All samples were isothermally heated up to the desired temperatures before cooling down to room temperature. Then, second heating runs were carried out with the same heating rates to establish the base lines.

The DSC thermograms presented in Figure 13a–e exhibited two opposite thermal events taking place at different temperatures. The onset temperatures for the first events were endothermic, appeared at lower temperatures in the range between 545 °C to 616 °C, as presented in Figure 13. These endothermic peaks are related to T_g_, which make for unique features of metallic glassy alloys. At this temperature, the solid-amorphous that is extended from room temperature to T_g_ is transformed into liquid-amorphous without structural or compositional changes.

**Figure 14:**
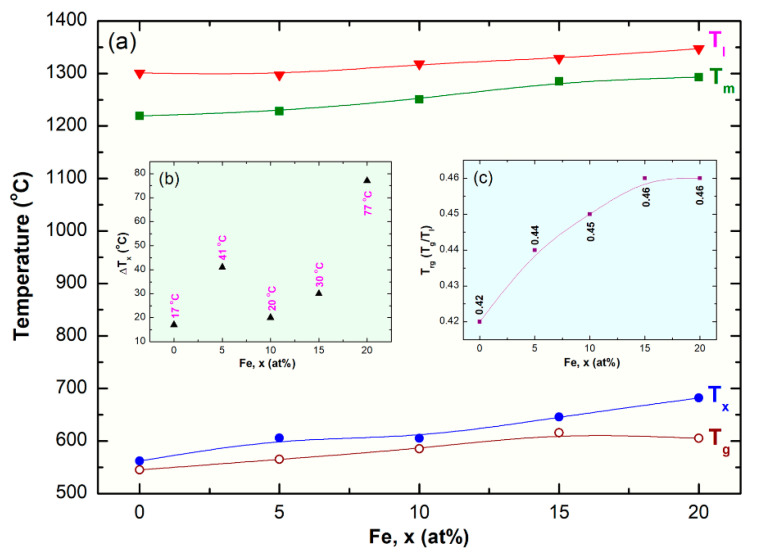
(**a**) Dependence of T_g_, T_x_, T_m_, and T_l_ on Fe molar fraction of SPS-(Co_75_Ti_25_)_100−x_Fe_x_ BMG system. The correlation between ΔT_x_ and T_rg_ and Fe content are elucidated in (**b**) and (**c**), respectively.

The second events, however, were characterized by sharp pronounced exothermic peaks, taking place at higher temperatures (T_x_) due to crystallization of the metallic glassy phase, as presented in Figure 13a–e. The area under the crystallization peaks refer to the ΔH_x_ of metallic glasses. Where T_g_ and T_x_ are usually used to describe the thermal stability of metallic glassy materials, ΔT_x_ is used to characterize their glass-forming ability (GFA). Wide ΔT_x_ indicates that the system has a good GFA. Depending on Fe molar fraction, metallic glassy (Co_75_Ti_25_)_100−x_Fe_x_ BMG system revealed very high T_g_ (545 °C to 616 °C), and T_x_ (562 °C to 682 °C), as displayed in Figure 13a–e and Figure 14a. This indicates that the system for all Fe concentration range possessed high thermal stability. Moreover, the wide values of ΔT_x_ before crystallization (17 °C to 77 °C), indexed in Figure 13a–e and Figure 14b, implies that the system is a good glass former.

All the DTA curves of SPS-(Co_75_Ti_25_)_100−x_Fe_x_ BMG system presented in Figure 13f–j display single endothermic events for each sample. Each peak contained obvious head and tail points, which are called melting (T_m_) and liquids (T_l_) temperature, respectively (Figure 13f–j). The onset temperature of both T_m_ and T_l_ increased slightly from 1219 °C and 1301 °C to 1393 °C and 1347 °C increasing the Fe molar fraction, as indexed in Figure 13f–j and Figure 14c.

In parallel to the ΔT_x_ parameter, the GFA of metallic glassy alloy systems produced by rapid solidification technique are described by a new parameter called reduced glass-transition temperature (T_rg_), which is equal to T_g_/T_l_. For good GFA systems prepared by rapid solidification technique T_rg_ should be larger than 0.5. Metallic classy (Co_75_Ti_25_)_100−x_Fe_x_ system in all ranges of Fe content had lower values, being in the range between 0.42 to 0.46, as illustrated in Figure 14c. As a result, the present metallic glassy system is a challengeable system that cannot be easily prepared by the conventional rapid solidification approach. Based on the thermodynamic parameters investigated in the present study, it can be concluded that Fe alloying element was beneficial to improve both thermal stability and GFA of (Co_75_Ti_25_)_100−x_Fe_x_ BMG system. This is demonstrated by the monotonical increase in T_g_, T_x_, ΔT_x_, and T_rg_ upon increasing the Fe content in the range between 5 at.% to 20 at.%, as indicated in Figure 14.

Table 4 summarizes some of thermodynamic parameters and magnetic properties of SPS-(Co_75_Ti_25_)_100−x_Fe_x_ BMG system.

## 4. Conclusions

Due to the poor GFA of CoTi-based alloy systems, it is very difficult to obtain glassy phases by rapid solidification approach. The present study has shown the possibility of employing a MA approach, using a high-energy BM method to fabricate new metallic glassy alloys of (Co_75_Ti_25_)_100−x_Fe_x_ (x; 0–20 at.%). Based on the results of this study, the following conclusions can be derived:

(1) The (Co_75_Ti_25_)_100−x_Fe_x_ system can be prepared successfully in a wide Fe concentration ranging from 0 to 20 at.%.
(2) The end product of the glassy phases obtained after 60 h of milling coexisted with a marginal volume fraction of nanocrystalline Fe powders.
(3) In MA-(Co_75_Ti_25_)_80_Fe_20_ system, a bcc-FeCoTi solid-solution phase was obtained after BM for 30 h. The powders of this obtained bcc-solid-solution were severely plastically deformed due to the effect of ball–powder–ball collisions, leading to the generation of intensive lattice imperfections composited of dislocations and point and lattice defects.
(4) Increasing the BM time enhanced the mechanically induced imperfections, leading to a solid-solution-to-amorphous phase transformation upon BM for 60 h.
(5) The as-fabricated (Co_75_Ti_25_)_80_Fe_20_ glassy alloy system revealed excellent GFA and good thermal stability, indicated by their wide ΔT_x_ and high T_x_ values.
(6) Based on the their wide ΔT_x_ before crystallizations and high T_x_, the as-fabricated powders were consolidated into nearly full dense (above 99.95%) bulk buttons, using SPS technique.
(7) The SPS consolidation step maintained the original short-range order structure after consolidation without experience of any partial crystallizations.
(8) The as-prepared metallic glassy (Co_75_Ti_25_)_100−x_Fe_x_ systems possesses good soft magnetic properties, indicated by high values of saturation magnetization (0.61 to 1.01 T), which increased with increasing Fe concentration.

## Figures and Tables

**Table 1:** Nominal and real compositions of as-prepared SPS-(Co_75_Ti_25_)_100−x_Fe_x_ systems.

**Nominal Composition (at.%)**							
Fe (x)	0	2	5	7	10	15	20
Co	75.00	73.5	71.25	69.75	67.50	63.75	60
Ti	25.00	24.5	23.75	23.25	22.5	21.25	20
**Real Composition, After Consolidation (at.%)**							
Fe (x)	0	1.96	5.15	7.01	9.98	14.99	20.14
Co	74.91	73.41	71.17	69.70	67.44	63.78	60.13
Ti	25.09	24.63	23.68	23.29	22.58	21.23	19.73

**Table 2:** EDS elemental analysis of as-consolidated (Co_75_Ti_25_)_80_Fe_20_ button.^1.^

Alloying Elements (wt.%)			
Zone	Co	Ti	Fe
I	63.14	17.08	19.78
II	62.98	17.10	19.92
III	62.94	16.98	20.08
IV	62.97	17.05	19.98
V	63.05	17.03	19.92
VI	62.91	16.96	20.13
VII	62.96	16.97	20.07
VIII	63.04	17.03	19.93

^1^ The analytical zones are shown in Figure 10a.

**Table 3:** EDS elemental analysis of as-consolidated (Co_75_Ti_25_)_100−x_Fe_x_ buttons.^1.^

Alloying Elements (wt.%)			
Zone	Co	Ti	Fe
I	78.75	21.25	-
II	78.64	21.36	-
III	78.72	21.28	-
IV	71.27	18.68	10.05
V	71.31	18.68	10.10
VI	71.29	18.71	10.00
VII	67.22	17.93	14.85
VIII	67.19	17.91	14.90
IX	67.21	17.96	14.83
X	62.98	16.97	20.05
XI	63.08	16.99	19.93
XII	63.04	17.01	19.95

^1^ The analytical zones are shown in Figure 11.

**Table 4:** Thermodynamic parameters and magnetic properties, measured for SPS-(Co_75_Ti_25_)_100−x_Fe_x_ BMG systems.

System	Temperature (°C)					T_rg_	B_s_ (T)
	T_g_	T_x_	ΔT_x_	T_m_	T_l_		
Co_75_Ti_25_	545	562	17	1219	1301	0.42	0.61
(Co_75_Ti_25_)_98_Fe_2_	548	571	23				0.70
(Co_75_Ti_25_)_95_Fe_5_	565	606	41	1228	1297	0.44	0.71
(Co_75_Ti_25_)_93_Fe_7_	568	609	41				0.72
(Co_75_Ti_25_)_90_Fe_10_	561	605	44	1251	1318	0.43	0.87
(Co_75_Ti_25_)_85_Fe_15_	616	646	30	1285	1329	0.46	0.94
(Co_75_Ti_25_)_80_Fe_20_	605	682	77	1293	1347	0.45	1.01
